# Large-area Scanning Probe Nanolithography Facilitated by Automated Alignment and Its Application to Substrate Fabrication for Cell Culture Studies

**DOI:** 10.3791/56967

**Published:** 2018-06-12

**Authors:** I-Ning Lee, Joseph Hosford, Shuai Wang, John A. Hunt, Judith M. Curran, William P. Heath, Lu Shin Wong

**Affiliations:** ^1^Manchester Institute of Biotechnology & School of Chemistry, University of Manchester; ^2^School of Engineering, University of Liverpool; ^3^School of Electrical and Electronic Engineering, University of Manchester; ^4^School of Science and Technology, Nottingham Trent University

**Keywords:** Bioengineering, Issue 136, scanning probe lithography, polymer pen lithography, automated alignment, parallelization, nanolithography, cell culture, stem cells, mesenchymal stem cells, cell-surface interactions

## Abstract

Scanning probe microscopy has enabled the creation of a variety of methods for the constructive ('additive') top-down fabrication of nanometer-scale features. Historically, a major drawback of scanning probe lithography has been the intrinsically low throughput of single probe systems. This has been tackled by the use of arrays of multiple probes to enable increased nanolithography throughput. In order to implement such parallelized nanolithography, the accurate alignment of probe arrays with the substrate surface is vital, so that all probes make contact with the surface simultaneously when lithographic patterning begins. This protocol describes the utilization of polymer pen lithography to produce nanometer-scale features over centimeter-sized areas, facilitated by the use of an algorithm for the rapid, accurate, and automated alignment of probe arrays. Here, nanolithography of thiols on gold substrates demonstrates the generation of features with high uniformity. These patterns are then functionalized with fibronectin for use in the context of surface-directed cell morphology studies.

**Figure:**
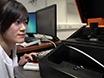


## Introduction

Progress in nanotechnology is dependent on the development of techniques capable of efficiently and reliably fabricating nanoscale features on surfaces.^1^,^2^ However, generating such features over large areas (multiple cm^2^) reliably and at relatively low cost is a non-trivial endeavor. Most existing techniques, derived from the semiconductor industry, rely on ablative photolithography to fabricate 'hard' materials. More recently, lithographic techniques derived from scanning probe microscopy (SPM) have emerged as a convenient and versatile approach for the rapid prototyping of nanoscale designs.^3^ SPM-based techniques are able to conveniently and rapidly 'write' any user-defined pattern. The most well-known of these is dip-pen nanolithography (DPN), pioneered by Mirkin *et al.*,^4^ where a scanning probe is used as a 'pen' to transfer a molecular 'ink' to the surface producing features in a fashion analogous to writing. Under ambient conditions, as a probe is scanned across a surface the 'ink' molecules are transferred to the surface *via* a water meniscus that forms between the probe and the surface (Figure 1). DPN thus allows the nanolithographic deposition of a wide range of materials, including 'soft' materials such as polymers and biomolecules.^5^ Related techniques using probes engineered with channels for fluid delivery, variously referred to as 'nanopipettes' and 'nano-fountain pens', have also been reported.^6^,^7^,^8^

The main obstacle to the wider application of SPM-derived lithography is throughput, as it requires an excessively long time to pattern centimeter-scale areas with a single probe. Early efforts to address this issue focused on the parallelization of cantilever-based DPN, with both 'one- dimensional' and 'two-dimensional' (2D) probe arrays being reported for the lithography of centimeter-sized areas.^5^,^9^ However, these cantilever arrays are produced through relatively complex multistep fabrication methods and are relatively fragile. The invention of polymer pen lithography (PPL) addressed this issue by replacing the standard SPM cantilevers with a 2D array of soft siloxane elastomer probes bonded to a glass slide.^10^ This simple probe setup significantly decreases the cost and complexity of patterning large areas, opening up nanolithography to a wider range of applications. This cantilever-free architecture has also been expanded to hard-tip soft-spring lithography,^11^ which provides a hybrid of soft elastomeric backing with hard silicon tips giving improved resolution in comparison to patterns produced using soft elastomer tips.

A crucial factor in the execution of these 2D array technologies is that the probe array must be exactly parallel to the surface substrate so that when lithography is utilized, all the probes come into contact with the surface simultaneously. Even a small misalignment can cause a large difference in feature size from one side of the array to the other, since some probes will come into contact with the surface earlier during the descent of the array, while others will come into contact later or not at all.^12^ Exact alignment is especially important with PPL due to the deformability of the soft elastomer probes, where the probes contacting the surface earlier will be compressed, leaving a larger footprint on the surface.

The early work on PPL employed purely visual inspection to guide the alignment process, using a camera mounted above the array to observe the deformation of the pyramidal probes as they were brought into contact with the surface.^10^ Alignment was judged by observing which side of the probes came into contact with the surface first, then adjusting the angle and repeating the procedure in an iterative manner until the difference in contact on each side of the probe was indistinguishable to the eye. As this alignment procedure relies on subjective visual inspection by the operator, reproducibility is low.

Subsequently, a more objective approach has been developed, consisting of a force sensor mounted beneath the substrate to measure the force applied upon contact of the probes on the surface.^12^ Alignment was thus achieved by adjusting the tilt angles to maximize the force exerted, which indicated that all the probes were simultaneously in contact. This method showed that alignment to within 0.004° of the surface parallel was possible. This 'force feedback levelling' has now been implemented into fully automated systems in two independent reports.^13^,^14^ Both use a triad of force sensors mounted either beneath the substrate or above the array and measure the amount of force exerted upon contact between the probe arrays and surface. These systems give high precision, reporting misalignments of ≤0.001° over a 1 cm length scale,^14^ or ≤ 0.0003°over 1.4 cm.^13^ These automated alignment systems also provide major savings in operator time and overall time taken to complete the lithography process.

One major application of high-throughput surface fabrication enabled by this technology is the generation of cell culture substrates. It is now well established that cell phenotype can be manipulated by controlling the initial interaction between cells and surface features, and that this can be enhanced at the nanoscale.^15^ Specifically, scanning probe lithography methods have been shown to be a facile method to produce a variety of nanofabricated surfaces for such cell culture experiments.^16^ For example, surfaces presenting nanoscale patterns of self-assembled monolayers and extracellular matrix proteins templated by PPL and DPN have been used to study the potential of nano-modified materials in material induced differentiation of stem cells.^17^

This protocol describes the utilization of a modified atomic force microscope (AFM) system that enables large-area PPL. We detail the detection of force using multiple force sensors as the means of determining probe-surface contact, together with an algorithm that automates the iterative alignment process. Subsequent functionalization of these patterns with the extracellular matrix protein fibronectin and the culture of human mesenchymal stem cells (hMSC) are described, as a demonstration of PPL-fabricated surfaces applied for cell culture.

## Protocol

### 1. Fabrication of the PPL pen array

1. To prepare the polydimethylsiloxane (PDMS) copolymer mixture:
  1. Add 10 µL of the platinum(0)-1,3-divinyl-1,1,3,3-tetramethyldisiloxane complex solution and 172 µL of 1,3,5,7-tetramethyl-1,3,5,7-tetravinylcyclotetrasiloxane to 250 g of (7-8 % *w/w* vinylmethylsiloxane)-dimethylsiloxane co-polymer. Mix these components thoroughly on a rotary mixer for 7 days to ensure homogenous mixing. CAUTION: platinum(0)-1,3-divinyl-1,1,3,3-tetramethyldisiloxane is toxic. Please read MSDS before working with this solution. Safety equipment must be worn while handling the chemical.
  2. Add 0.5 g of (25-35 % *w/w* methylhydrosiloxane)-dimethylsiloxane co-polymer to a 1.7 g portion of mixture from step 1.1.1 in a weighing boat and mix thoroughly with a spatula.
  3. Degas this mixture by transferring it to a vacuum desiccator and exposing the mixture to low pressure (200 mTorr, 0.3 mBar) for 20 min until all the gas bubbles have dissipated.
2. Place a 13 x 13 mm glass slide in a plastic screw-topped vial filled with 20 mL 2-propanol, then place the vial in an ultrasonic bath for 10 min to remove any large debris. Wash the slides by submerging the slides in fresh 2-propanol (100 mL) and dry under a stream of nitrogen gas.
3. Place a silicon master^18^ into a 4 cm diameter petri dish and add sufficient degassed PDMS prepolymer mixture (from step 1.1) until fully covered. Typically, 100 µL is required for a 20 x 20 mm master. Place the master with the prepolymer mixture in a vacuum dessicator. Degas the polymer for a further 5 min to remove any gas bubbles formed during the transfer of the mixture. The O_2_ plasma treatment of the glass slides (step 1.4 below) should be performed while the degassing is taking place.
4. Treat the glass squares with O_2_ plasma (600 mTorr) at maximum RF power for 1 min to remove any organic contamination and to generate a uniform oxide layer on the glass for adhesion of the elastomer.^19^ Use the plasma treated slides immediately in the next step.
5. Carefully place the square glass slide (from step 1.4) over the prepolymer on the master (from step 1.3) with the plasma-cleaned side facing down. Gently press down the glass slide onto the silicon master to remove any trapped air and to ensure a uniform film of PDMS is sandwiched between the master and the slide.
6. Place the sandwiched PDMS array from above step in a petri dish with the silicon master at the bottom (*i.e.*, with the back of the glass slide facing upwards) and place the dish in an oven at 70−80 °C for 24−48 h to thermally cure the PDMS.
7. Remove the cured array from the oven and allow to cool for 15 min, then with a razor blade carefully remove any excess PDMS from the back and sides of the glass slide and use a stream of dry nitrogen to blow away any loose PDMS debris. Note: Take care not to scratch the silicon master with the razor blade, as this may damage the non-stick coating.
8. Wedge a razor blade into the corner of the array at a depth of 1 mm and carefully pry the array apart from the master. Perform this action in a single continuous lifting action; do not allow the arrays to fall back onto the master.
9. Carefully cut and scrape away 0.5 mm of the PDMS at the edges of the array with a razor blade. Use a stream of dry nitrogen to blow away any loose PDMS debris. Note: It may be easier to perform this trimming step under stereoscope or a magnifying glass. Take care not to scratch the silicon master with the razor blade, as this may damage the non-stick coating.

### 2. Array preparation and substrate mounting

1. Generate a hydrophilic surface on the probe array by O_2_ plasma treatment:
  1. Place the PPL pen array in a petri dish into plasma chamber then apply vacuum to 600 mTorr. Switch on the plasma generator (maximum setting) for 30 s.
  2. Release the vacuum, remove the array and check its hydrophilicity by dropping 20 µL of deionized water onto the array and observing whether there is even spreading of the water across the surface. If this does not occur, subject the array to a second round of plasma treatment. Afterwards, dry the array thoroughly with a stream of dry nitrogen gas.
2. Using double-sided carbon tape, attach the array onto the middle of the probe holder. Mount the probe holder onto the AFM kinematic holder (Figure 2).
3. To load the PPL array with 16-mercaptohexadecanoic acid (MHA) ('inking'):
  1. Prepare 1 mM 16-mercaptohexadecanoic acid (MHA) solution by dissolving 8.6 mg in 30 mL ethanol in a tube and placing it in an ultrasonic bath for 10 min to fully dissolve the compound. CAUTION: 16-mercaptohexadecanoic acid is toxic. Please read MSDS before working with this solution. Safety equipment must be worn while handling the chemical.
  2. Using a micropipette, deposit 20 µL drop of the MHA solution on the array. Avoid contact of the pipette tips with the arrays. Allow it to spread throughout the array, then allow the ethanol to evaporate under ambient conditions. NOTE: The PPL array can alternatively be inked after the alignment has taken place.^10^
4. ​Once the MHA solution has dried, mount the probe holder with the PPL array onto the AFM.

### 3. Preparation of gold substrates for PPL.

1. Gold substrates can either be purchased, or made in-house by thermal or electron beam deposition, and are constructed of a 2 nm titanium adhesion layer followed by 20 nm of gold on a glass or silicon wafer.^18^
2. Where necessary, clean the substrates by oxygen plasma treatment using the parameters described in step 1.4.
3. Place the gold substrate in the middle of the AFM sample stage and secure with adhesive tape around the borders of the substrate (Figure 2). Adjust the stage to the correct height as indicated in the manufacturer's operating instructions using the *z*-axis controller.

### 4. Automatic alignment of pen array

1. Open and run the stage controller setup program (SetupNSF.exe) on the computer to reset ('zero') all axes and angles to a pre-calibrated zero point, then use the stage *x*/*y*-axis controller console to move the substrate to the desired alignment/printing location. For optimal results, the substrate should be placed near the center of the stage, between the stage's force sensors. NOTE: In some models of computer, the *x*/*y*-axis controller USB signal may interfere with that from the *z*-axis controller. If this occurs, disconnect the *x*/*y*-axis controller USB cable after this step. It should then be reconnected after the alignment procedure (step 4.7).
2. Switch the stage release lever to release the sample stage and activate the triad of force sensors as indicated by the AFM manufacturer's instructions. Allow the force sensors to equilibrate for at least 15 min. For optimal results, allow 30-50 min.
3. Increase the z-axis height to bring the array into close proximity with the substrate by visually observing the probe array and surface. NOTE: The closer the array is to the surface, the fewer iterations are required for the alignment process, thus saving time.
4. Open/run the Automatic Alignment program (Auto Alignment v16.exe) and enter relevant alignment parameters into the program.
  1. Enter the desired 'Angle Step' parameter value, typically 0.15°. This parameter is the offset angle from the 'optimum' angle for each axis that is determined by the program. Set this parameter between 0.1 and 0.2°, as angles lower than this range do not result in a clearly detectable force difference upon approach of the probes to the surface. NOTE: Software accepts values in millidegrees *(i.e.*, 1 x 10^-3 ^°). For example, for 0.15°, users should input '150.'
  2. Entering the desired Coarse Step' parameter value, typically 0.6 µm. This parameter is the z-axis step size used by the stage as it approaches the probes in the initial rough alignment. Set this parameter between 0.2 and 1 µm. Larger step sizes decrease the time taken for the alignment process but reduce the accuracy of the alignment, and increase the wear on the probes. NOTE: Software accepts values of coarse steps in micrometers. For example, for 0.6 µm users should input '0.6'.
  3. Enter the desired 'Fine Step' parameter value, typically 0.2 µm. This parameter is the z-axis step size used for fine adjustment of the optimum alignment. For most applications, set this parameter between 0.1 and 0.4 µm. Larger value step sizes will decrease the amount of time taken for the alignment process but reduce the quality of the alignment. NOTE: Software accepts values of fine steps in micrometers. For example, for 0.2 µm, users should input '0.2.'
  4. Configure the 'Excel file path' and attach an unmodified copy of the provided spreadsheet template file by using the 'folder' icon, navigating to the file location, and pressing 'OK'. This file contains the raw and calculated data that is used to determine the optimum stage tilt angles of the stage.
5. Open/run the AFM control software. Navigate to the spectroscopy component of this program by clicking the 'spectroscopy' button (according to the manufacturer's instructions), and set the AFM scan head z-axis to oscillate by 10 µm over 100 ms, with a pause time of 250 ms, then to retract 10 µm over 100 ms with a pause time of 250 ms (Figure 3).
6. As the AFM head is oscillating, click the 'start' button of the alignment software to begin the automated alignment process. When the program is running, the software is writing and reading data in the file described in step 4.4.4. NOTE: The alignment takes between 30 min and 3 h depending on the initial stage position set in step 4.3 and software configuration that were entered in step 4.4. CAUTION: The stage controller consoles are still active during the alignment process - do not use them during the alignment process as it will interfere with the alignment.
7. When the alignment finishes, the green light box 'Alignment Completed' on the alignment software (from step 4.4) will be lit. When this occurs, click the 'STOP' button on the user interface to end the alignment process.
8. Inspect the graphs in the spreadsheet template file for a correlation between recorded data points and the line fit that is generated by the software. See representative results for examples of a good correlation, with typical R^2^ values of > 0.99. If alignment is unsuccessful, replace the probe array with a newly prepared array (step 2) and repeat the alignment (step 4.4).
9. Move the stage upwards in the *z*-axis using the stage controller console for that axis. The stage should be moved in 500 nm increments until contact can be observed from the top view camera of the AFM. Contact between the array and substrate can be observed as a 'white dot' of high contrast at the apex of the individual probe pyramids.
10. At this point, click the 'stop' button on the AFM control software to stop the spectroscopy program from step 4.5. This will retract the array by 10 µm, therefore leaving 10 µm of possible z-axis extension. Check the image from the top view camera of the AFM to ensure that the probes are not in contact with the substrate.

### 5. Polymer pen lithography (PPL)

1. Navigate to the lithography component of the control software by clicking the 'lithography' button on control software. Choose the *z*-modulation operating mode and import a raster (bitmap) or vector image that will be used as the lithography pattern. In order to generate the features shown in the representative results, use a bitmap consisting 20 x 20 black pixels (see supplemental material), corresponding to the lithography of a grid of 20 x 20 dots per probe on the PPL array.
2. Enter the lithography parameters into the 'Pixel Graphic Import' window of the AFM controller software.
  1. Configure the 'Size' of the pattern to be generated, *e.g.*, 40 µm in length and width. These parameters indicate the width and length over which the image in the bitmap will be scaled. To generate features shown in the representative results, use a width and length of 40 µm in both dimensions.
  2. Set the 'Origin' of the pattern to be generated at 25 µm on both *x* and *y* axis. These parameters determine the center of the image relative to the center of the AFM *x*/*y*-axes. Set these parameters to avoid the region of the surface where the probes were in contact during the alignment process.
  3. Set the printing 'Parameters'. These values determine how the probes are to be extended (i.e. brought into contact with the surface) in response to each pixel in the bitmap image.
    1. Select from the drop-down menu 'Modulation Abs Z Pos' and 'Simplify to' two layers. This mode instructs the AFM to extend the probes by an absolute distance determined by only two results, either 'Black (0)' or 'White (1)' fields.
    2. Set the values in the 'Black (0)' and 'White (1)' fields to 5 and -5 µm, respectively. These values determine the distance the probes should be moved in response to a black or white pixel on the bitmap image and are typically set between 3 and 5 µm for 'Black' (*i.e.*, extend probes downwards by that distance relative to the zero point of that axis) and -3 to -5 µm for 'White' (*i.e.*, withdraw the probes upwards by 3 to 5 µm relative to the zero point). NOTE: These representative distances assume that a 5 µm extension results in the probes coming into contact with the surface and hence the generation of a feature, while a 5 µm withdrawal lifts the probes away from the surface resulting in no contact. *Z*-extension affects feature size by determining the extent of probe contact with the surface, greater extensions result in the probes being pressed further into the surface, resulting in larger features.^10^
    3. Click the 'OK' button to implement these settings and close the window.
3. Enter the 'pause time' in the lithography window of the AFM control software, typically 1 s. This setting determines the length of time the probes remain in the extended 'Black' position, which is typically set between 0.1 and 10 s. NOTE: Longer pause times result in larger feature sizes due to the larger amount of MHA transported to the gold surface. Further details on controlling the size of features generated can be found in other reports.^20^
4. Prepare the atmospheric control enclosure.
  1. Lower the atmospheric isolation chamber onto the AFM and open/run the manufacturer-supplied atmospheric control software (MHG_control.exe).
  2. Set the atmospheric control software to maintain a relative humidity of 45%, a temperature of 25 °C, and an atmosphere exchange 'Flow rate' of 500 mL by entering these values into the software. Click 'Use' to implement the settings. The atmospheric control module will then begin to pump humidified air into the chamber. NOTE: Higher humidity levels result in larger feature sizes due to the formation of a larger water meniscus generated between pen arrays and surface.^21^ This value is typically set between 40 and 60%. The flow rate is typically set between 300 and 500 mL. Larger flows allow the desired humidity level to be reached more rapidly but is less accurate. The representative results use a flow rate of 500 mL for initial generation of humidity and is decreased to 300 mL upon reaching the desired humidity, to maintain an accurate and stable level during lithography.
5. Once desired humidity is obtained, start the lithographic sequence by pressing the 'start' button on the software interface.
6. Upon completion of the lithography, use the z-axis stage controller console to move the substrate away from the array by retracting the stage by 500 µm. Then remove the atmospheric isolation chamber from its mount.
7. Switch the stage release lever to lock the sample stage and deactivate the force sensors, as indicated by the AFM manufacturer's instructions, then remove the substrate from the stage.

### 6. Pattern visualization

1. Patterns can be visualized using one of the following methods, lateral force scanning probe microscopy or chemical etching.
2. Scan the patterned surface on AFM with lateral force mode using contact mode cantilever to examine the features nondestructively. NOTE: Lateral force microscopy can be used as a nondestructive method of viewing the features produced by polymer pen lithography; however, using this method, only a relatively small area can be visualized (typically 100 x 100 µm).
3. Since the deposited MHA can act as an etch resist, chemical etching can be used to remove the gold from the non-patterned areas. The resulting unetched areas can then be visualized by optical microscopy, meaning a wide area can be viewed at once.^18^ NOTE: Substrates that are etched in this way cannot then be used for the subsequent cell culture experiments described below.
  1. Separately, prepare aqueous solutions of 40 mM thiourea, 27 mM iron(III) nitrate and 100 mM hydrochloric acid. Prepare the etchant by mixing 5 mL of each of these three solutions. Freshly mix prior to each use.^22^ CAUTION: thiourea, iron(III) nitrate and hydrochloric acid are toxic. Please read MSDS before working with this solution. Safety equipment must be worn while handling the chemical.
  2. Transfer the patterned substrate into a petri dish and pipette sufficient etchant solution into the dish to cover the surface of the substrate (typically 10 mL). Keep the substrate submerged for 4-5 min to etch 15 nm of gold (at an approximate rate of 3 nm/min).
  3. When etching is completed, remove the substrate and thoroughly rinse with water and dry with a stream of nitrogen. NOTE: The completion of etching process is determined by the thickness of gold surface obtained (step 3.1) associated with the etching rate specified (step 6.2.2).
  4. Inspect the etched gold features under bright field optical microscopy. The remaining gold features that remain should appear corresponding to the pattern that was printed (from step 5.2). If the entire surface appears homogeneous, this indicates that a significant amount of gold remains and the etching step (following step 6.2.2) should be repeated for 1-2 min.

### 7. Pattern functionalization with fibronectin

1. Immerse the patterned substrates into a solution of 1 mM (11-mercaptoundecyl)hexa(ethylene glycol) solution in ethanol for 1 h. Wash the substrate three times with ethanol and dry thoroughly under a stream of nitrogen. This step passivates the unpatterned gold areas. CAUTION: (11-mercaptoundecyl)hexa(ethylene glycol) is toxic. Please read MSDS before working with this solution. Safety equipment must be worn while handling the chemical.
2. Submerge the substrates in a 10 mM Co(NO_3_)_2_ aqueous solution for 5 min. Next, remove the substrates from the solution, wash three times with ultrapure water, and dry under a stream of dry nitrogen. CAUTION: Co(NO_3_)_2_ is toxic. Please read MSDS before working with this solution. Safety equipment must be worn while handling the chemical.
3. Immerse the substrate in a 50 µg/mL solution of fibronectin in phosphate buffered saline (PBS) and incubate at 4 °C for 16 h. Wash the substrate three times with PBS, then dry the substrate under a stream of dry nitrogen. NOTE: Fibronectin is bound to MHA-functionalized areas through chelation of Co(II) by the terminal carboxylic acid groups on the MHA. Fibronectin then binds to Co(II) *via* its collagen-binding domain.^23^
4. If desired, visualize the surface-bound fibronectin by labelling it with fluorescent antibodies:
  1. Apply a 2 mL solution of 1:100 unconjugated rabbit anti-fibronectin primary antibody in 5% (w/v) of bovine serum albumin (BSA) in PBS to the surface and incubate at 4 °C for 16 h. Aspirate the supernatant and wash three times with PBS.
  2. Submerge the substrate in a 2 mL solution of fluorescently conjugated anti-rabbit secondary antibody (at the manufacturer's specified dilution, 2 drops/mL) in 5% (w/v) of BSA in PBS, cover in tin foil, and incubate at room temperature for 1 h. Aspirate the supernatant and wash three times with PBS.
  3. Record the epifluorescence microscopy images of the features using a fluorescence microscope according to the manufacturer's instructions, with an excitation filter set to 594 nm.

### 8. Cell culture on nanofabricated surfaces

1. Prepare a suspension of well characterized hMSCs that are between the 4^th^ and 6^th^ passage.^24^
  1. Suspend a confluent flask of cells by rinsing once with 10 mL PBS, dissociate adhered cells by adding 5 mL of trypsin/EDTA into the T75 tissue culture flask, and incubate the flask in a humidified chamber at 37 °C supplemented with 5% CO_2 _for up to 5 min until 90% of cells are detached from surface.
  2. Subsequently, add 6 mL of fresh culture media containing 10% fetal calf serum (FCS) into the flask and briefly rinse the flask with the media added. Transfer cell suspension into a 15 mL centrifuge tube and centrifuge at 400 g at 25 °C for 5 min.
  3. Remove the supernatant and resuspend cell pellet in 3 mL of fresh culture media.
  4. Count the cell density using a hemocytometer^25^ and adjust the density of the cell suspension to 2 x 10^4^ cells/mL by the addition of an appropriate volume of culture media.
2. Seed the cells onto substrates at a density of 10^4^ cells/cm^2^.
  1. Cut the substrate into 1 x 1 cm^2^ with diamond scribe and place it in a well in 12-well tissue culture plate.
  2. Pipette 2 mL of cell suspension in culture media (from step 8.1.4) into the well and incubate in a humidified chamber at 37 °C supplemented with 5% CO_2_ for 24 h.
3. After cell growth on the patterns, analyze the extent of cell attachment and spreading by immunofluorescence:
  1. Remove media and wash substrates once with PBS. Fix cells with 2 mL of a solution of 4% paraformaldehyde in PBS (pre-warmed to 37 °C) for 20 min in fume hood and wash three times with PBS. CAUTION: Paraformaldehyde is toxic. Please read MSDS before working with this solution. Safety equipment must be worn while handling the chemical.
  2. Permeabilize the cells with 2 mL of a solution of 0.5% detergent (see **Table of Materials**) in PBS for 15 min, then wash three times with PBS.
  3. Submerge the substrate in 2 mL of a solution of unconjugated rabbit anti-fibronectin primary antibody at dilution of 1:100 with 5% (w/v) BSA in PBS and incubate at 4 °C for 16 h, then wash three times with 0.1% (v/v) Tween 20 in PBS (PBST).
  4. Subsequently submerge the substrate in 2 mL of a solution of fluorescently conjugated anti-rabbit secondary antibody (diluted with 5% (w/v) BSA in PBS at the manufacturer's specified dilution, 2 drops/mL), cover in tin foil and incubate at room temperature for 1 h, then wash three times with 0.1% PBST.
  5. To label actin filaments, submerge 2 mL of fluorescently conjugated phalloidin at a dilution of 1:250 in PBS, cover in tin foil and incubate at 4 °C for 30 min then wash three times with PBS.
  6. Simultaneously stain cell nuclei and mount the substrate by applying a drop of mounting medium containing DAPI and cover with a coverslip.
4. Visualize cells using a fluorescence microscope according to the manufacturer's instructions, with excitation filters of 365 nm for nuclei (DAPI), 488 nm for F-actin and 594 nm for fibronectin.

## Representative Results

To check whether the automated alignment had been successful, the graphs plotted from the alignment data (in the spreadsheet from step 4.8) were examined. Where the alignment process had been successful the two plots, corresponding to the angle by which the sample stage has been tilted along the θ and φ axes, showed a series of rising and descending data points. In each of the plots, two linear fits of the data points showed a well-defined intersect "peak" indicating the maximum *z*-extension and the corresponding angle at which alignment was achieved (Figure 4A** and 4B**). This process is repeated four times (*i.e.*, twice for each axis) and plotted as a set of four coordinates. The intersection of each pair of coordinates thus shows the overall optimum angles (Figure 4C).^13^ In cases where the alignment was not successful, it can be observed that their corresponding θ and φ angle plots do not give good quality linear fits, or do not intersect (Figure 5). Such failed alignments are typically as a result of the arrays being improperly trimmed or mounted to the probe holder (steps 1.7, 1.8, and 2.2). In these cases, the arrays were discarded and a new one prepared and mounted (steps 1 and 2), and the alignment process repeated (step 4).

Upon successful alignment and lithography with MHA by PPL, patterned gold substrates were then imaged using lateral force microscopy to examine whether deposition had taken place. A larger area examination of the printed surfaces was also conducted by optical microscopy of the substrates after etching of the gold not protected by the deposited thiol (Figure 6 and Figure 7). However, the etched patterns cannot be used for further functionalization and should only be used to confirm patterning on representative samples of a batch of printed surface substrates. If the etched patterns show blank areas corresponding to individual pens (Figure 8), this result indicates that the production of probe arrays has not been done successfully, and that some probes are damaged or missing. This inhomogeneity of the probes may be due to the use of an old master where the perfluorinated coating has worn away (step 1.3), resulting in some probes being torn away when the array is separated from the master. In these cases, a new master should be used. The result may also be due to the presence of air bubbles trapped between the glass backing and the master (step 1.5), or if the probe array was not cleanly separated from the master after curing (step 1.8).

Florescent microscopy images of the fibronectin functionalized surfaces incubated hMSCs were also collected (Figure 9). In general, all substrates were stable within the *in vitro* culture environment and the cells adhered and adapted their morphology to the printed patterns in case of smaller isolated 20 x 20 array of features.

**Figure:**
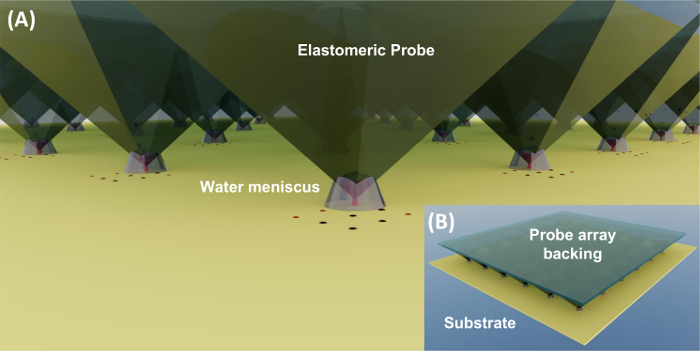

**Figure 1. Schematic representation of polymer pen lithography showing molecular ink transport *via* a water meniscus on probe tip.** (A) Side view and (B) top view of the polymer pen array indicate that when the probe array and surface substrate are fully aligned, all the probes come into contact with the surface simultaneously, resulting in parallelized lithography. Please click here to view a larger version of this figure.

**Figure:**
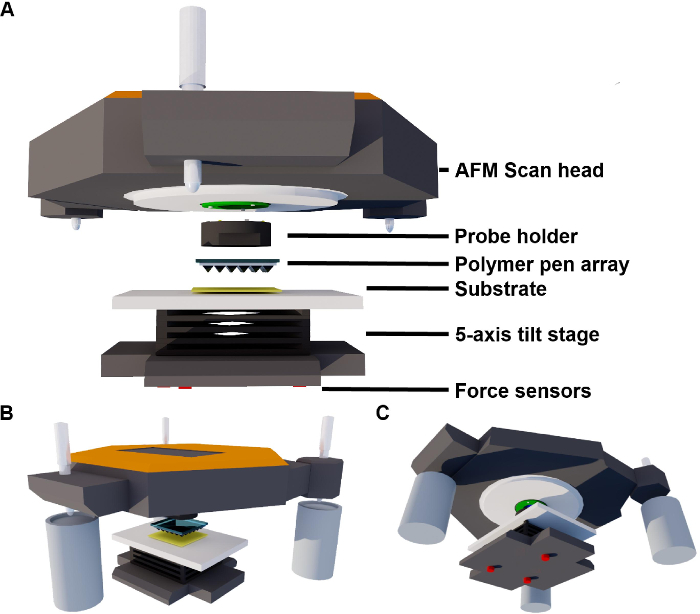

**Figure 2. Schematic diagram of polymer pen lithography set up.** (A) Expanded side view of experimental set up where the prepared probe array is attached to probe holder and mounted to AFM scanner. The substrate is placed on the stage, below which are located the three force sensors. (B) A representation of the assembled instrumentation, showing the AFM scan head relative to the sample stage. (C) Bottom view showing force sensor location. Please click here to view a larger version of this figure.

**Figure:**
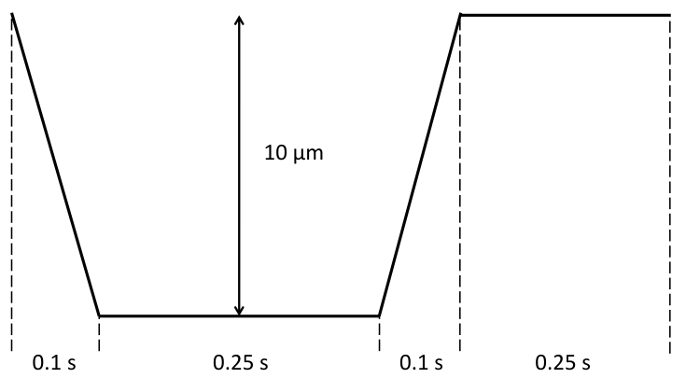

**Figure 3. Schematic representation depicting the spectroscopy program for the alignment procedure.** The AFM scanner is set to move the probes toward sample by a distance of 10 µm within 100 ms, held at position for 250 ms, followed by a retraction of 10 µm within 100 ms, and then held for 250 ms at the retracted position. The motion is then repeated throughout the alignment process. Please click here to view a larger version of this figure.

**Figure:**
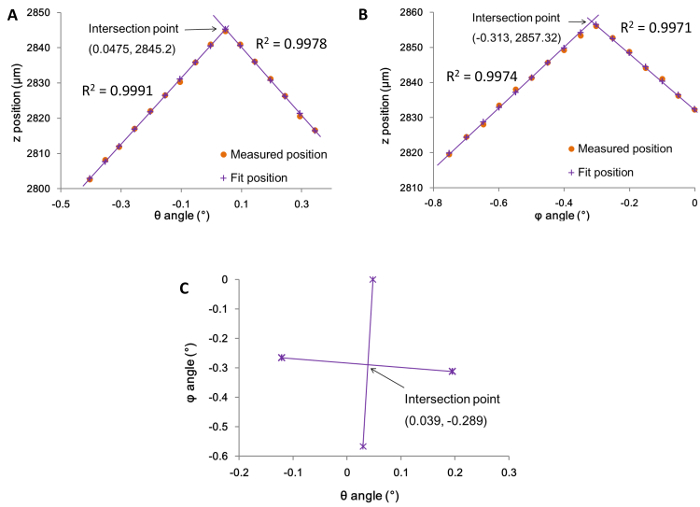

**Figure 4. Graphs illustrating a successful alignment.** Graphs of z position against the tilt angles (A) θ and (B) φ for a successful alignment, where ● indicates the actual values measured and **+** indicates the best fit with the least-squares method. (C) Graph of φ against θ fitted angles with the four points where the maximum z-position was reached. The intersection point marked is the final optimum tilt angle across both axes. Please click here to view a larger version of this figure.

**Figure:**
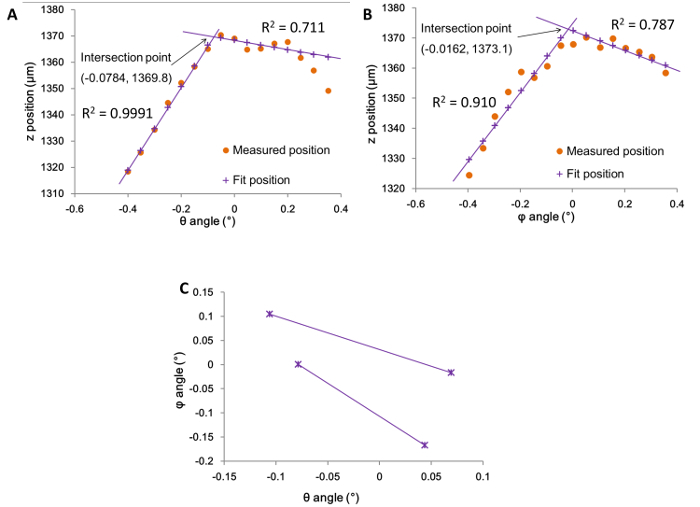

**Figure 5. Graphs illustrating an unsuccessful alignment.** Graphs of z position against the tilt angles (A) θ and (B) φ for an unsuccessful alignment, where ● indicates the actual values measured and **+** indicates the best fit with the least-squares method. (C) Graph of φ against θ fitted angles with the four points where the maximum z-position was reached. No clear optima or intersection point are observed and therefore the optimum alignment angles are not resolved. Please click here to view a larger version of this figure.

**Figure:**
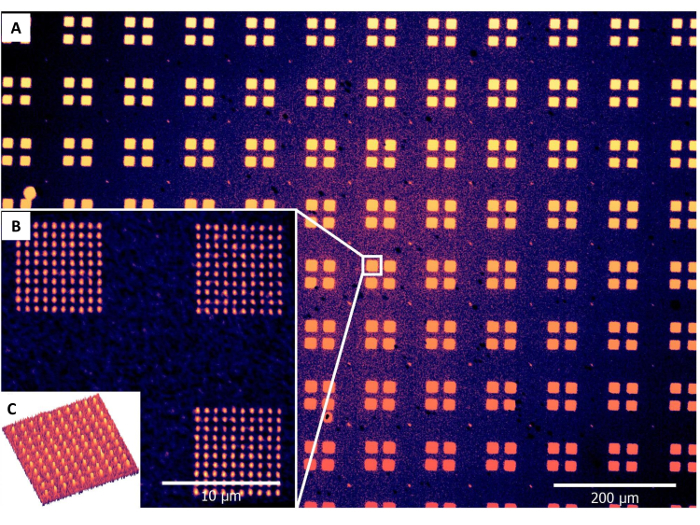

**Figure 6. Illustrative optical microscopy and atomic force microscopy images of gold substrates that were patterned with MHA by the aligned PPL arrays and then etched.** (A) and (B) are sequentially magnified optical microscopy images of the etched patterns; (C) is an AFM topography image of a single grid of patterns. Please click here to view a larger version of this figure.

**Figure:**
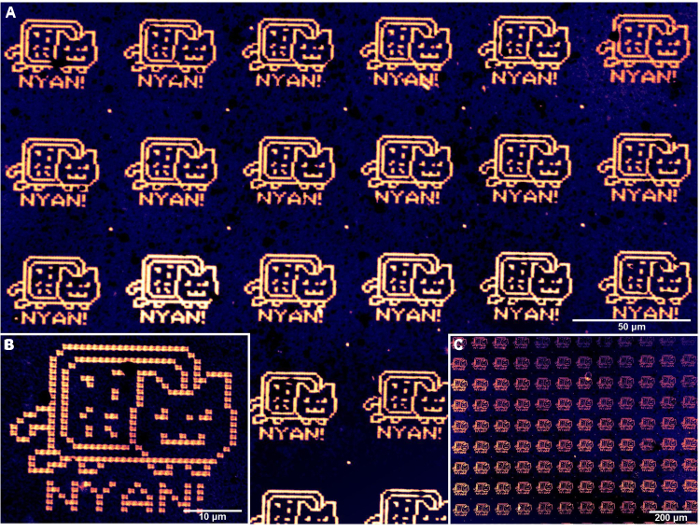

**Figure 7. Illustrative optical microscopy images of gold substrates that were patterned with MHA by the aligned PPL arrays and then etched.** (A) and (B) are sequentially magnified optical microscopy images of etched patterns and (C) is a lower magnification image that shows large area homogeneous patterns. Please click here to view a larger version of this figure.

**Figure:**
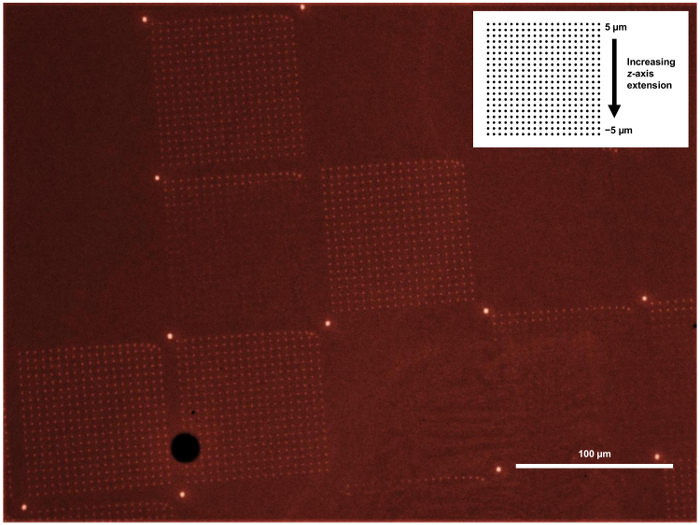

**Figure 8. Illustrative optical microscopy image of a gold substrate that was unevenly patterned with MHA and then etched.** The intended patterns (shown in the inset) were repeating grids of 20 dot lines arranged in 20 lines, with every two lines produced by increasing the *z*-axis extension by 1 µm (ranging from 5 to -5 µm). It can be seen that in some areas no patterns are generated, due to missing probes in those locations. In the areas where only two lines of dots are produced, this result indicates that a probe is present but it is not of the same height as the fully functioning probes, so only deposit features when the array is extended to the full *z*-axis distance. In this image, the contrast has been deliberately altered to enable observation of the partially printed areas. Please click here to view a larger version of this figure.

**Figure:**
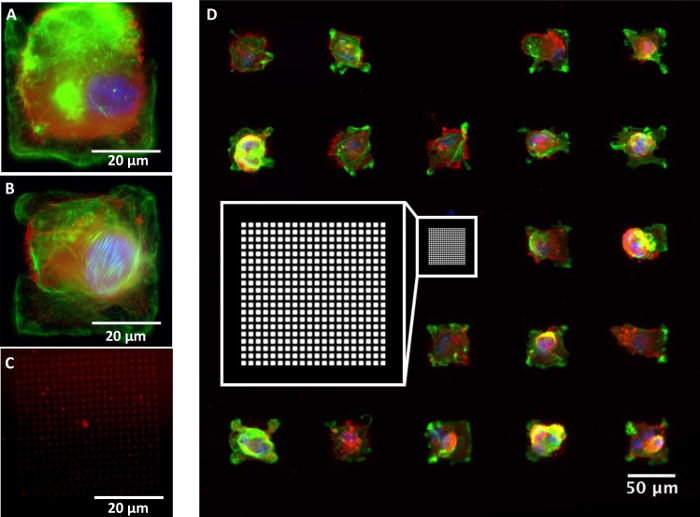

**Figure 9. Epifluorescence microscopy images of hMSCs cultured on the fibronectin arrays templated by PPL.** (A) and (B) are high magnification images showing individual cells. (C) Shows an example pattern of the fibronectin array without an adherent cell and (D) is a wide field image of the cells cultured in a grid arrangement (a schematic of the printed pattern is also shown in the inlay). The cells are stained to show fibronectin (red), F-actin (green) and cell nuclei (blue). Please click here to view a larger version of this figure.

## Discussion

This protocol serves to provide users with a convenient methodology to rapidly carry out nanolithographic patterning with high uniformity and controllable feature size over large (cm^2^) areas. Substrates bearing these large area nanopatterns can then be further elaborated for a variety of applications. One major application of this technology is in the generation of nanofabricated surfaces for cell-surface interaction studies. This report shows some illustrative examples of cell culture on these materials, demonstrating control of hMSC morphology by nanofabricated substrates.

The key enabler of this protocol is the automation of the alignment procedure (step 4) that allows highly uniform and high-throughput production of features on surfaces, down to nanoscale resolution, which enables the rapid turnover of cell culture experiments. The polymer pen lithography carried out using this alignment algorithm is able to generate nanoscale features within approximately 30 min. The reproducibility and accuracy of automated alignment, and thus the uniformity of the patterned features, is however critically dependent on the quality of the probe arrays that are produced (step 1 and 2). Any flaws in their preparation that result in blunt, broken or missing probes; such as trapped air bubbles (step 1.5) or improper separation of the probes from the master (step 1.8) can result in inaccurate alignment and poor quality lithography.

This reported method shares a limitation in common with other alignment methods that rely on force feedback. The accurate determination of when the probes are in contact with the surface is constrained by the need to account for background vibrations caused by the ambient environment and the movement of the sample stage. In general, the sensors have a force sensitivity in the µN regime (2 µN in this case), but the alignment algorithm is designed to only register a force of at least 490 µN as definitive contact between the probes and the surface, in order to avoid any 'false positives' resulting from background noise.^13^ Thus, this method tends to produce large features (1-2 µm) since the probes must extended a large distance on the *z*-axis (with a consequent higher force) in order to register contact. In order to compensate, smaller features can be generated by reducing the *z*-axis distance travelled during the lithography step (*e.g.*, entering the 'Black' setting in step 5.2.3.2 as 3 µm instead of 5 µm).

Nevertheless, even with this limitation, the automation algorithm is able to address a critical aspect in the application of parallelized scanning probe lithography methods, as alignment was previously the most time demanding and imprecise step in the implementation of these techniques. This automation now shifts the rate-limiting step of the fabrication process from the alignment to the lithographic writing itself. While this protocol demonstrates the application of this alignment procedure to PPL, the framework could be applied to a number of SPL techniques such as lipid-DPN^26^ and matrix-assisted lithography^27^ as well as potential future catalytic probe systems.^28^

## Disclosures

The alignment algorithm and software were developed by, and are proprietary to, the University of Manchester. It is available for download at http://www.click2go.umip.com/.
